# Alarm Pheromones and Chemical Communication in Nymphs of the Tropical Bed Bug *Cimex hemipterus* (Hemiptera: Cimicidae)

**DOI:** 10.1371/journal.pone.0018156

**Published:** 2011-03-30

**Authors:** H. Christoph Liedtke, Kajsa Åbjörnsson, Vincent Harraca, Jette T. Knudsen, Erika A. Wallin, Erik Hedenström, Camilla Ryne

**Affiliations:** 1 Chemical Ecology, Department of Ecology, Lund University, Lund, Sweden; 2 Limnology, Department of Ecology, Lund University, Lund, Sweden; 3 Chemistry, Department of Natural Sciences, Engineering and Mathematics Mid Sweden University, Sundsvall, Sweden; New Mexico State University, United States of America

## Abstract

The recent resurge of bed bug infestations (*Cimex spp.*; Cimicidae) and their resistance to commonly used pesticides calls for alternative methods of control. Pheromones play an important role in environmentally sustainable methods for the management of many pest insects and may therefore be applicable for the control of bed bugs. The tropical bed bug, *Cimex hemipterus*, is a temporary ectoparasite on humans and causes severe discomfort. Compared to the common bed bug, *Cimex lectularius*, little is known about the chemical signalling and pheromone-based behaviour of the tropical species. Here, we show that the antennal morphology and volatile emission of *C. hemipterus* closely resembles those of *C. lectularius* and we test their behavioural responses to conspecific odour emissions. Two major volatiles are emitted by male, female and nymph *C. hemipterus* under stress, (*E*)-2-hexenal and (*E*)-2-octenal. Notably, nymph emissions show contrasting ratios of these compounds to adults and are further characterized by the addition of 4-oxo-(*E*)-2-hexenal and 4-oxo-(*E*)-2-octenal. The discovery of this nymph pheromone in *C. hemipterus* is potentially the cause of a repellent effect observed in the bio-tests, where nymph odours induce a significantly stronger repellent reaction in conspecifics than adult odours. Our results suggest that pheromone-based pest control methods developed for *C. lectularius* could be applicable to *C. hemipterus*, with the unique nymph blend showing promising practical properties.

## Introduction

As their generic name suggests, the tropical bed bug, *Cimex hemipterus*, is a hematophageous insect, parasitizing on human hosts living in equatorial regions [1]. Together with its temperate relative the common bed bug, *Cimex lectularius*, they have a substantial economic impact via litigations, costly control efforts and loss of business [2]. With infestations currently on the rise [3]–[5] developing effective management strategies is of utmost importance. Health precautions and development of resistance against common pesticide agents [6]–[8] has warranted research into alternative, semiochemical-based control methods. Exploiting alarm pheromones for this purpose is of interest and a recent study on *C. lectularius* showed promising results: a widespread tool for insect control has been the use of desiccant dusts, which damage the water proofing cuticular lipids of pests and ultimately causes death by desiccation. The addition of synthetic *C. lectularius* alarm pheromone to such dusts by Benoit et al. [9] compelled these insects to leave their refuges more readily and increased their overall motor activity. This resulted in increased transpiration and rate of contact with the desiccant dusts and made this control method up to three times more effective.

*C. lectularius* is primarily distributed in the northern hemisphere and has become the model organism both for bed bug pest control as well as bed bug pheromone research [1], [8], [10]–[13]. Conversely, *C. hemipterus* has been largely overlooked presumably as it is not a nuisance in the western world. However, it is a domestic pest in Florida [1], [14] and there are records of recent infestations in the U.K. [15] and Australia [16], well outside their preferred latitude. These records suggest a geographic range expansion and the two species have been reported to thrive as mixed infestations [17]. This is cause for concern because pheromone-based control methods designed for *C. lectularius* may not prove effective for combating *C. hemipterus* infestations, as species-specific blend composition and ratios are often very important in chemical communication systems [12], [18]. Almost nothing is known about the chemical ecology of *C. hemipterus* and therefore this study investigates three major constituents of chemical communication: the morphology of the main receptive organ (the antennae), the volatiles emitted under stress and the behavioural responses induced by these volatiles in conspecifics.

Silver-staining and scanning electron microscopy (SEM) of *C. hemipterus* extremities has indicated a chemosensory nature of the most distal antennal segment [19]. By more detailed SEM imaging of the external antennal anatomy, we hereby aim to generate a sensilla map for *C. hemipterus* comparable to that of an existing map for *C. lectularius* produced by Steinbrecht and Müller [20]. This will allow investigations into the claims of Singh et al. [19] that the tropical species (*C. hemipterus*) has double the number of chemosensitive sensilla compared to *C. lectularius* as well as exhibiting sexual dimorphism, a characteristic not evident in the common bed bug [20].

*C. hemipterus* has been described as odourless [1], but this is unlikely to be true (*pers. obs.*). Of ten compounds constituting the *C. lectularius* nest odours, (*E*)-2-hexenal and (*E*)-2-octenal secreted from dorsal abdominal glands in nymphs and metathoracic glands in adults have been identified as important signalling compounds, acting primarily as an alarm pheromone, eliciting dispersal behaviour in conspecifics [21], [22]. Two recently described nymph compounds, 4-oxo-(*E*)-2-hexenal and 4-oxo-(*E*)-2-octenal [23], are also biologically active in this species, deterring males during mating attempts [24]. These four aldehydes are ubiquitous communication agents within Hemiptera and their role as deterrents and defensive chemicals is strongly conserved in this order [18], [25]–[27]. As the tropical bed bug is a close taxonomic relative of the common bed bug, we predict that these volatiles will be major constituents in the alarm emissions of *C. hemipterus* as well and will elicit similar repulsive behaviours as those observed in *C. lectularius* [21], [26]. By identifying the constituents of volatile emissions using coupled gas chromatography mass spectrometry (GC-MS) and assessing behavioural responses to these volatile emissions using bioassays, our findings aim to contribute to the development of population control agents which have thus far focused exclusively on *C. lectularius* [9].

## Materials and Methods

Ethic guidelines: All biotests were performed on insects (Hemiptera), thus ethic guidelines do not apply.

### Insects

*C. lectularius* and *C. hemipterus* were reared and maintained for several generations at Lund University (Sweden). All insects were kept in a climatic chamber with L12∶D12, constant T = 25°C+/−2°C and RH = 70%+/−0.5%. Insects were fed *in vitro* every ten days on fresh, defibrinated chicken blood as described by Montes et al. [28]. Insects used in bioassays were wild caught individuals from the Muhaka village (32 km south-southwest of Mombasa, Kenya 4°19.5′S 39°31.5′E) and behavioural studies were performed on site within days of their capture.

### Sensory mapping

Sensilla located on the most distal antennal segment of *C. hemipterus* were mapped using photographs taken at high magnifications on a JEOL JSM-5600LV scanning electron microscope operated at 15 kV. To prepare the specimens, males and females were dried first in 70% then in 99.5% ethanol (Solveco AB, Sweden) for 24 h per treatment. The antennae were then mounted onto aluminium stubs and sputter coated with gold∶palladium alloy (60∶40) in a Polaron E 5400 high-resolution sputtering unit. Sensilla types were identified using morphological descriptions by Steinbrecht and Müller [20].

### Collection of bed bug emissions

The volatiles emitted by live bed bugs were isolated by headspace collection with Tenax and analysed by thermal desorption. All collections were carried out in a climatic chamber (∼25°C, ∼60%RH). Although data on the blend ratios of *C. lectularius* alarm pheromones already exists [13], [29], collections were repeated here to standardize the methods and equipment used, allowing for an accurate comparison between species.

Collections were carried out on groups of four individuals at a time, with males, females and late instar nymphs of both species separated into individual Teflon tubes (7 cm long, 0.5 cm in diameter). The insects were allowed to recover from the invasive handling for ∼30 min before continuing the experimental procedures. Each tube was then fitted with a 40 mg Tenax GR filter (mesh size 60–80, Alltech, USA) on one end to purify the incoming air and a 10 mg Tenax GR filter (mesh size 60–80, Alltech, USA) on the other end to absorb emitted volatiles. Controls (tubes with no insects) were run in parallel to screen for background compounds and possible contaminations. To stimulate the emission of alarm pheromones by the insects, carbon dioxide was passed through each tube for ten seconds at a low flow rate (not to physically agitate the bed bugs) shortly before the start of odour collections [1]. Each setup was then connected to a suction pump via the 10 mg filter with the flow adjusted to 20 ml/min and the volatiles were collected over two hours. Trapped volatiles were eluted with 200 µl of hexane (≥98%, Merck KGaA, Germany) and 100 ng of ethyl hexadecanoate was added as internal standard. Before analyses, all samples were concentrated four to five times under a stream of pure nitrogen.

### Analysis of bed bug emissions

Extracts were analyzed on a gas chromatograph (GC; Hewlett-Packard 6890 Series) equipped with a non-polar capillary column (HP-5MS; 30 m long, 0.25 mm in diameter and 0.25 µm film thickness, Agilent Technologies, USA) coupled to a mass spectrometer (MS; Hewlett-Packard 5973 Mass Selective Detector). The injector temperature was 225°C and the injector was splitless. Temperature was programmed for 3 min at 40°C followed by a gradual increase of 8°C/min to reach a final temperature of 230°C for 5 min. Retention times and GC peak areas were obtained by integration of peaks using Agilent ChemStation software (Agilent Technologies, USA). All sample chromatograms were compared to their corresponding controls and any background compounds were subtracted from the sample prior to analysis.

The identities of the compounds proposed by Adam and NIST98 databases were confirmed with retention times and spectra of co-injected synthetic reference compounds. The syntheses of two commercially unavailable compounds found in the head space collections, 4-oxo-(*E*)-2-hexenal and 4-oxo-(*E*)-2-octenal, followed the protocol described by Moreira and Miller [30] and their Fourier transform infrared spectra (FTIR) are provided as a supporting figure (Figure S1).

### Quantification and statistical analysis of bed bug emissions

Peak areas were quantified against the added internal standard. In addition, the four most abundant compounds, (*E*)-2-hexanal, (*E*)-2-octenal, 4-oxo-(*E*)-2-hexanal and 4-oxo-(*E*)-2-octenal were quantified more accurately using dose-response curves calibrated by injecting six known quantities (0.01, 0.1, 1.0, 10 100 and 1000 ng/µl; with 100 ng of internal standard added) of synthetic reference compounds into the GC-MS. The resulting quantities of compounds collected were divided by four to get a per-individual measure and then expressed in nanograms per sample per hour. One-way ANOVA and Tukey's post hoc analysis were used to compare absolute quantities and ratios of (*E*)-2-hexenal and (*E*)-2-octenal in males, females and nymphs and the ratio of 4-oxo-(*E*)-2-hexenal and 4-oxo-(*E*)-2-octenal (only present in nymphs) was analyzed with Student's T-test. To better fit the assumptions for ANOVA and normalizing the distribution of the residuals, all ratios were arcsine square root transformed. All data analysis was carried out using SPSS v17.0 software (SPSS Inc., USA).

### Bioassays

Behavioural experiments were performed at the International Centre of Insect Physiology and Ecology (ICIPE) field station located near Muhaka village. The experiments were performed under ambient climate conditions (25–28°C; 70–90%RH) and in dimmed light. *C. hemipterus* were collected over two days, from five houses in Muhaka village. Males, females and nymphs of different sizes were immediately sorted and stored in separate jars. The insects were kept for at least two days before the experiments commenced. Nymphs that had moulted into adults, as well as eggs oviposited during this time were removed and sorted accordingly. As *C. lectularius* feed at intervals of seven to ten days *ad libitum* [31], the collected insects were not fed during the five-day experimental period.

Twenty males were placed in a glass vial with a screw-on cap containing 800 µl of distilled hexane, for 60 min, to obtain whole body extracts. The insects were then removed and the solvent retained for use in the bioassay. The same protocol was applied for females and late instar nymphs and an extract containing both males and females (ten of each) was also prepared. Between experiments, the extracts were stored below 0°C to reduce the risk of degradation (oxygenation) and evaporation of active volatile compounds.

Petri dishes (17 cm in diameter) were reused as arenas for the behavioural studies and were sterilised with 70% ethanol and thoroughly aired for more than 30 min before each use. Odour sources were prepared by pipetting 10 µl of the different extracts onto small pieces of filter paper (approx. 0.5 cm^2^), allowing 5 min for the hexane solvent to evaporate completely before the commencement of each trial. Pure hexane controls were also prepared in the same manner. The arena was then divided into two equal halves and the odour source was placed 0.5 cm from the edge in one of the two halves (odour section). A single bed bug was thereafter introduced into the middle of the Petri dish and its position relative to the two halves was monitored every five seconds over two minutes, by an observer positioned approximately 0.5 m away. Bio-tests were restricted to two minutes to ensure sufficient emission rates of compounds from the filter paper throughout the experiment [32].

The number of visits to the odour section was counted and analysed with two-way ANOVA, against the null-hypothesis of equal visits between the two halves. Bed bugs on the line were not counted and the Petri dishes were rotated 90° clockwise between trials, to reduce the influence of observer presence on bed bug behaviour. In total, twenty males, twenty females, and twenty nymphs ranging from third to fifth instar were tested individually for each extract. No individual was reused and all extracts, including controls, were tested daily in random order with replications added each day.

## Results

### Sensory mapping

**Figure 1:**
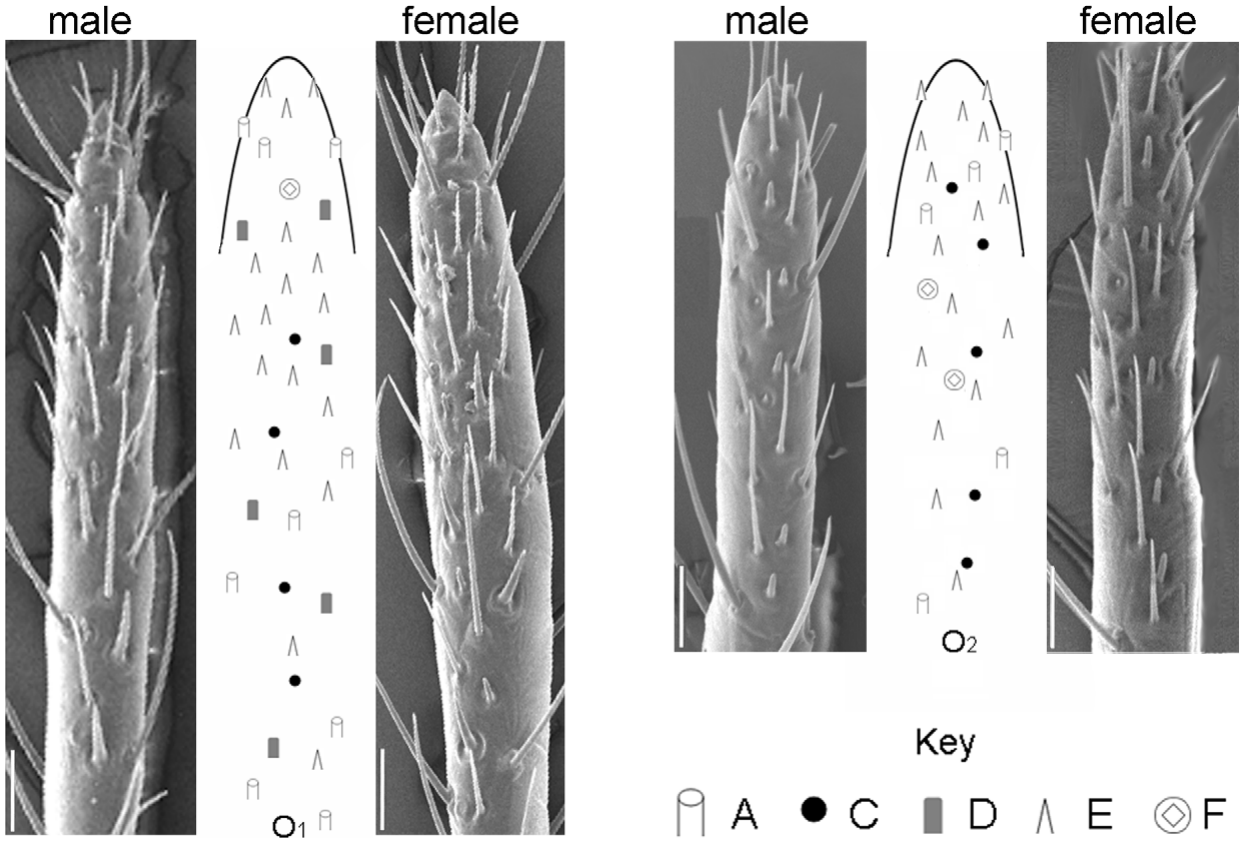
Sensilla distribution on terminal antennal segment of *C. hemipterus*. Schematic maps of sensillum distribution on the inner (O_1_) and outer (O_2_) surfaces of the terminal antennal segment of *C. hemipterus*, flanked by Scanning Electron Microscopy (SEM) photographs of the corresponding regions in males and females to demonstrate a lack of sexual dimorphism. The map was derived from analysis of SEM images of 12 male and 9 female antennae. The position and relative distances between individual sensilla are roughly to scale, leaving room for minute individual variation and scale bars represent 20 µm. For *C. lectularius* comparison, see Steinbrecht and Müller [20].

Twelve male and nine female *C. hemipterus* antennae were examined and different sensilla types were identified based on their external morphology as described by Steinbrecht and Müller [20]. A total of five sensilla types were recorded. All five types were mapped, but most attention was given to the chemosensitive sensilla types C and D, which are of importance in chemical detection in *C. lectularius* [10], [20], [22], [24]. These were arranged in two fields on the inner (O_1_) and outer (O_2_) surface of the distal tip of the terminal antennal segment in both males and females (Figure 1; Table 1). O_1_ contains a greater overall number of sensilla than O_2_ and extends considerably further proximally on the terminal antennal segment. Flattened, bare surfaces sparsely speckled with mechanoreceptors (not shown on Figure 1), separated the two olfactory regions. There was a high consistency in both the number and patterning of sensilla between individuals as well as between sexes (Table 1). Subtypes for A and E sensilla described for *C. lectularius* were not separated as this requires transmission electron microscopy [19], [20].

**Table 1:** Number of sensilla in male and female *C. hemipterus*.

	Sensilla Type				
	A	C	D	E	F
**Male** *(n = 12)*					
O_1_	7–9	4	6	12–18	1
O_2_	3–5	5	0	10–17	2
**Female** *(n = 9)*					
O_1_	6–8	4	6	16	1
O_2_	3–6	5	0	14–17	2

Summary of the total number of each type of sensilla present on the olfactory regions (O_1_ and O_2_) located on the distal tip of the terminal antennal segment of *C. hemipterus* for males and females, determined from SEM photographs. Lettering of sensilla types corresponds to Steinbrecht and Müller [20] with type A: bristle, C: grooved peg, D: smooth peg, E: trichoid and F: immersed cone sensilla.

### Analysis of bed bug emissions

The pheromone components in the head space of 32 *C. hemipterus* samples (nine female-, twelve male- and eleven nymph samples) and 41 *C. lectularius* samples (thirteen female-, seventeen male- and eleven nymph samples) were collected and their gas chromatograms were analysed (Figure S2). After screening against the negative controls, nine resulting compounds were detected, all of which are fatty acid derived compounds with the exception of an irregular terpene, geranylacetone. Two compounds, (*E*)-2-hexenal and (*E*)-2-octenal were recovered in the greatest amounts in all 73 samples. Two additional compound, 4-oxo-(*E*)-2-hexenal and 4-oxo-(*E*)-2-octenal were detected in all nymph samples of both species but were absent in head space collections of adults (Table 2; Figure S2). Low quantities, with GC peak heights less than 1% of the largest peak, of (*Z*)-2-hexenal, (*Z*)-2-octenal, (*E*)-2-hexenol, (*E*)-2-octenol and geranylacetone were detected in some, but not all samples. The first four compounds are believed to be breakdown products of, or involved in the biosynthetic pathway of (*E*)-2-hexenal and (*E*)-2-octenal [18], [33] and geranylacetone is known to be present in human emanations [34]. They are thus unlikely to be bed bug alarm pheromones and this investigation will focus exclusively on the four most prominent compounds.

**Table 2:** Quantification of compounds in head space collections of *C. hemipterus* and *C. lectularius*.

*Cimex hemipterus*							
	Retention time (min)	Female (*n = 9*)		Male (*n = 12*)		Nymph (*n = 11*)	
		Mean	SD	Mean	SD	Mean	SD
(*E*)-2-hexenal	6.68	0.90*^abcd^*	1.14	1.10*^abcd^*	1.43	0.48*^abc^*	0.36
4-oxo-(*E*)-2-hexenal	9.06	n. d.		n. d.		0.32*^ab^*	0.31
(*E*)-2-octenal	11.19	0.38*^ab^*	0.35	0.88*^abcd^*	0.86	2.03*^bcd^*	1.58
4-oxo-(*E*)-2-octenal	12.91	n. d.		n. d.		0.02*^a^*	0.02

*Cimex lectularius*							
	Retention time (min)	Female (*n = 13*)		Male (*n = 17*)		Nymph (*n = 11*)	
		Mean	SD	Mean	SD	Mean	SD
(*E*)-2-hexenal	6.68	6.91*^e^*	4.55	2.29*^bcd^*	3.15	0.26*^ab^*	0.13
4-oxo-(*E*)-2-hexenal	9.06	n. d.		n. d.		0.51*^abcd^*	0.35
(*E*)-2-octenal	11.19	6.54*^e^*	3.95	3.01*^cd^*	3.19	2.30*^d^*	1.24
4-oxo-(*E*)-2-octenal	12.91	n. d.		n. d.		0.37*^ab^*	0.23

Mean quantities (in ng/individual/hour) and standard deviation of the four most prominent compounds detected in headspace collections of *C. hemipterus* and *C. lectularius* females, males and nymphs. Different letters denote significant differences at p<0.05 (ANOVA; *F*_7,78_ = 6.031; *P*<0.001, followed by Tukey's post hoc test). n. d. not found or below detection level.

The variance for the quantities of individual compound emitted was high and only *C. lectularius* females produced significantly greater quantities of (*E*)-2-hexenal and (*E*)-2-octenal than their *C. hemipterus* counterparts (ANOVA; *F*_7,78_ = 6.031; *P*<0.001). No differences in the quantities of male and nymph emissions were statistically supported (Table 2).

**Figure 2:**
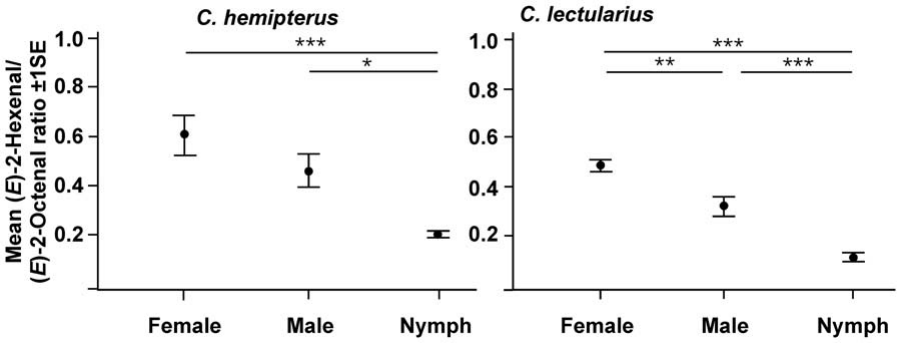
Aldehyde ratios in head space collections of *C. hemipterus* and *C. lectularius*. Mean (*E*)-2-hexenal∶(*E*)-2-octenal ratios present in the head space collections of *C. hemipterus* and *C. lectularius* females, males and nymphs. Asterisks correspond to statistical differences measure by Tukey's post hoc test with * *P*<0.05, ** *P*<0.01 and *** *P*<0.001. There is no significant difference when comparing groups between species (ANOVA; *F*_2,67_ = 0.031; *P* = 0.970).

An analysis of (*E*)-2-hexenal∶(*E*)-2-octenal ratios revealed that in *C. hemipterus*, (*E*)-2-hexenal is the more prominent constituent in the adult blends but the reverse is true for nymphs. *C. hemipterus* emitted (*E*)-2-hexenal and (*E*)-2-octenal at mean ratios of 59∶41 (female), 45∶54 (male) and 20∶80 (nymph; Figure 2). In comparison, *C. lectularius* emitted these compounds in mean ratios of 52∶48 (female), 68∶32 (male) and 11∶89 (nymph; Figure 2) yet these ratios were not significantly different to their *C. hemipterus* female, male and nymph counterparts (ANOVA; *F*_2,67_ = 0.031; *P* = 0.970). All differences in blend ratios within species however, were statistically significant, except between *C. hemipterus* females and males (*P* = 0.253; Figure 2).

In nymphs, the ratios of 4-oxo-(*E*)-2-hexenal to 4-oxo-(*E*)-2-octenal differs significantly between species (*t*_20_ = 12.6, *P*<0.001), with approximate 4-oxo-(*E*)-2-hexenal∶4-oxo-(*E*)-2-octenal ratios of 91∶9 and 53∶47 in *C. hemipterus* and *C. lectularius* respectively (Table 2).

### Bioassays

**Figure 3:**
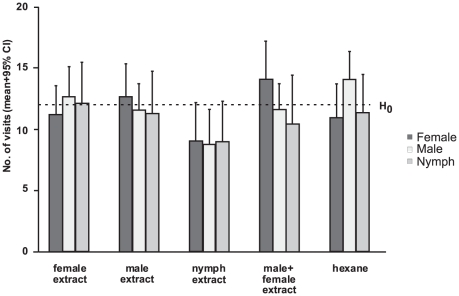
Behavioural responses to conspecific whole body extracts in *C. hemipterus*. Number of visits to the odour-containing area by either males, females or nymphs of *C. hemipterus* to whole body extracts of males (20), females (20), last instar nymphs (20), or a mix of males and females (10+10) recorded every fifth second of observation during two minutes. The dotted line represents the null hypothesis (50%).

All behavioural responses to treatments were normally distributed (Kolomogrov-Smirnoff *Z*<0.814, *P*>0.522, *N* = 20) and there was a significant treatment effect (ANOVA; *F*_[4]_ = 2.81; *P* = 0.026). Nymph odour repelled all tested insects (hexane-nymph extract: *P* = 0.05, while the responses of males, females and nymphs to all other extracts did not differ from the control (Figure 3).

## Discussion

The tropical bed bug, *Cimex hemipterus* shows strong similarities both in antennal morphology and alarm pheromone composition to its close relative, the common bed bug *C. lectularius*. Nymphs have an inversed alarm pheromone aldehyde ratio to that of adults and their pheromones are distinguished further by the addition of two unique oxo-aldehydes. Like in *C. lectularius* [24], this unique nymph blend elicits strong repellent reactions in conspecifics, a response which may be exploited for pest control.

Scanning electron microscopy of *C. hemipterus* antennae has revealed that this species possesses an extremely reduced number of chemosensitive sensilla compared to other hematophagous insects. The same is true for *C. lectularius* and has been explained as an adaptation to their close parasite-host association [22], [35], [36]. *Cimex hemipterus* shows little individual variation and no sexual dimorphism in sensilla number and patterning and thus a reliable map was generated. The total number, distribution and types of sensilla housed by *C. hemipterus* correlate strongly to the patterning of *C. lectularius* antennae [20]. In both species, chemo-sensitive sensilla are located on the tips of the most distal segment of the antenna and are clustered to form two olfactory regions, confined to the inward- (O_1_) and outward- (O_2_) facing surfaces [20]. In *C. lectularius*, the olfactory regions are equipped with six different sensillum types (nine including sub-types; [20], [22]), the presence of five of which could be confirmed for *C. hemipterus*. The presence of the sixth type, a placoid sensillum, could not be confirmed or rejected and requires higher resolution SEM photography to be detected. In *C. lectularius*, two sensillum types, the smooth- and grooved- pegs (types D and C) are sensitive to alarm pheromone constituents, the former hosting receptors for (*E*)-2-hexenal and (*E*)-2-octenal, 4-oxo-(*E*)-2-hexenal and 4-oxo-(*E*)-2-octenal [10] and the latter housing additional receptors for 4-oxo-(*E*)-2-hexenal and 4-oxo-(*E*)-2-octenal [24]. The number and patterning of these two sensillum types is extremely conserved in this genus and in this respect, the sensillum map for *C. hemipterus* published here is close to identical to that for *C. lectularius* printed in 1976 [20].

The inter-species similarity and lack of sexual dimorphism described above contradicts previous findings by Singh et al. [19]. The authors of that study inferred that *C. hemipterus* have more densely bristled antennae than *C. lectularius* [20] (showing higher counts for both olfactory and mechanical sensillum) and females host one and a half times more C and D sensilla than males. Their result is peculiar, as the uniformity in antennal morphology of the two species evident in our study coincides well with similarity in other anatomical and life history traits, relevant to their chemical ecology; both species have similar body sizes and longevity, live in large mixed-sex colonies, feed on the same hosts and practice a nocturnal lifestyle [1]. Furthermore, antennal dimorphism is often associated with the use of sex pheromones and because no such pheromones are apparent in this genus [13], the sexual homogeneity described here is reasonable.

The composition of the volatiles emitted by *C. hemipterus* closely resembled that of *C. lectularius* as well, with (*E*)-2-hexenal and (*E*)-2-octenal being the predominant constituents in both species [13], [18], [21], [26]. With the exception of female *C. lectularius* emitting larger quantities of the aldehydes than their *C. hemipterus* counterparts (which may explain why the latter has previously been described as odourless [1]), no differences in pheromone quantities is evident. It is important however, to draw attention to the high variance in the data. This is likely to be an artefact of individual variation in health, size of pheromone glands or extent of alarm response. Blend ratios on the other hand are a relative measure for each individual sample and will thus be given more weight in this discussion.

Nonetheless, the strong pattern of homogeneity persists when looking at the ratios at which the two species emit (*E*)-2-hexenal relative to (*E*)-2-octenal. This discovery has promising practical as well as economic implications: the use of this blend of aldehydes has proven to increase the effectiveness of experimental control method for *C. lectularius* [9] and therefore the same blend should be applicable for combating tropical bed bug- as well as mixed- infestations.

As is the case for *C. lectularius* [13], [23], adult and nymph *C. hemipterus* emit differing aldehyde blend ratios, with (*E*)-2-octenal being a more dominant component in nymph than adult emissions. This nymph-specific signature is strengthened by the presence of two extra compounds, 4-oxo-(*E*)-2-hexenal and 4-oxo-(*E*)-2-octenal, found exclusively in the head space of late instar nymphs and not in adults. The presence of these oxo-aldehydes was recently discovered in extracts of dorsal abdominal glands of the common bed bug [23] and here we show that these compounds also exist as volatiles in the emissions of nymphs of this species. Furthermore, we also show that the same oxo-aldehydes are present in the head space of juvenile *C. hemipterus*, once again allowing parallels to be drawn between the two species. 4-Oxo-(*E*)-2-hexenal is likely to be the dominant ratio component in the nymphs of both bed bug species, however the mean ratios differ, suggesting possible species-specificity. Harraca et al. [24] showed that *C. lectularius* grooved peg sensilla are sensitive to 4-oxo-(*E*)-hexenal as well as 4-oxo-(*E*)-2-octenal, but the latter only in high concentrations. Nonetheless, it can be concluded that nymph pheromone blends divert strongly from adult blends in two aspects; nymphs emit a reversed (*E*)-2-hexenal∶(*E*)-2-octenal ratio and contain two additional compounds, 4-oxo-(*E*)-2-hexenal and 4-oxo-(*E*)-2-octenal.

The bioassay emphasizes the importance of this adult-nymph pheromone divergence and substantiates the biological relevance of this finding. Nymph extracts induce a stronger repellent reaction in conspecifics than adult extracts, which implies that individuals can perceive the differences in nymph and adult pheromone composition. Similar behavioural responses have been described for *C. lectularius*, where both 4-oxo-(*E*)-2-hexenal and the nymph-characteristic aldehydes ratio act as anti-aphrodisiacs towards advancing males [24], possibly to prevent harmful traumatic insemination [31]. This study goes one step further and shows that the avoidance behaviour is not restricted to males as females and other nymphs are also repelled by nymph extracts. Nymph secretions may therefore act as alarm pheromones in aggregations of males, females and nymphs in general as well as signal immaturity to males specifically. Such multi-functionality is becoming increasingly more apparent in bed bugs and it is evident that the originally simplistic definition of an alarm pheromone is becoming more complex [10], [12], [13], [24]. Again, this result has important practical implications too: Benoit et al. [9] used an adult blend ratio in combination with desiccant dusts to target adult bed bugs and a nymph blend ratio to target nymphs in separate pest control trials. Here we show that such a division is unnecessary and a late instar nymph pheromone can be used to target bed bugs of all life stage, which is a more realistic scenario. We even expect an increase in effectiveness to using the adult blend, seeing as our bioassays showed that the nymph blend elicited a stronger reaction in adults than the adult blend did.

We have shown that the external morphology of *C. hemipterus* antenna closely resembles that of *C. lectularius* with no evidence of sexual dimorphism. The ratio of the two predominant volatiles emitted by these two bed bugs under stress, (*E*)-2-hexenal and (*E*)-2-octenal, are also highly conserved across species. Furthermore, we have shown that nymph volatile emissions are characterized by an inversed aldehyde ratio and two additional components, 4-oxo-(*E*)-2-hexenal and 4-oxo-(*E*)-2-octenal were, in this study, only found in the immature stages of these species. Interestingly, the ratio at which these two oxo-aldehydes are emitted appear to be species specific. Finally, we have shown that high concentrations of nymph pheromone induced a strong repellent effect on conspecifics of all life-stages and sexes. With increased infestation rates in mind [5], our findings have important implications for the development of an alarm pheromone-based pest control method that could target both species of bed bug.

## Supporting Information

Figure S1 **Infrared spectra of natural and synthetic odour components.** Infrared spectra of (*E*)-2-hexenal, (*E*)-2-octenal, 4-oxo-(*E*)-2-hexenal and 4-oxo-(*E*)-2-octenal from *C. lectularius* extracts and synthetic reference compounds. Gas Chromatography-Fourier transform infrared spectroscopy (GC-FTIR) analyses were carried out on extracts and reference compounds using a GC (Agilent Technologies 7890A) equipped with a polar capillary column (FactorFour, VF-23ms; 30 m long, 0.25 mm inner diameter and 0.25 µm film thickness), coupled to a FTIR (Termo Fisher Nicolet 6700 FT-IR). The injector temperature was 250°C and the injector was splitless. The temperature was programmed at 50°C for 0 min, followed by a gradual increase of 10°C/min to reach a final temperature of 230°C for 10 min. IR data for the compounds: 4-oxo-(*E*)-2-hexenal: IR (vapour phase): ν (cm^−1^): 2987, 2818, 27301, 1712, 1106, 1048, 980 and 4-oxo-(*E*)-2-octenal: IR (vapour phase): ν (cm^−1^): 2967, 1715, 1102, 1073, 980. IR (neat): ν (cm^−1^): 2958, 2933, 2873, 1711, 1172, 1049.
(TIF)

Figure S2 **Gas chromatograms of** ***C. lectularius*** **and** ***C. hemipterus*** **and mass spectra of nymph compounds.** Representative GC-traces of head space samples from A) *C. lectularius* and B) *C. hemipterus* with mass spectra of a) 4-oxo-(*E*)-2-hexenal, b) 4-oxo-(*E*)-2-octenal. 4-Oxo-(*E*)-2-octenal showed a M+ of 112, 113 from ^13^C and a base peak of 83 plus fragments of 55 and 57, and a M+ of 140, a base peak of 111 and fragments of 55, 83 and 85 respectively. Their identity was confirmed using synthetic reference compounds which produced the same GC-FTIR (Figure S1) and GC-MS spectral information.
(TIF)
