# Supplementary figures and images for: Alarm Pheromones and Chemical Communication in Nymphs of the Tropical Bed Bug Cimex hemipterus (Hemiptera: Cimicidae)
Source: PLoS One. 2011 Mar 30;6(3):e18156. doi: 10.1371/journal.pone.0018156 (PMC3068171; doi:10.1371/journal.pone.0018156)

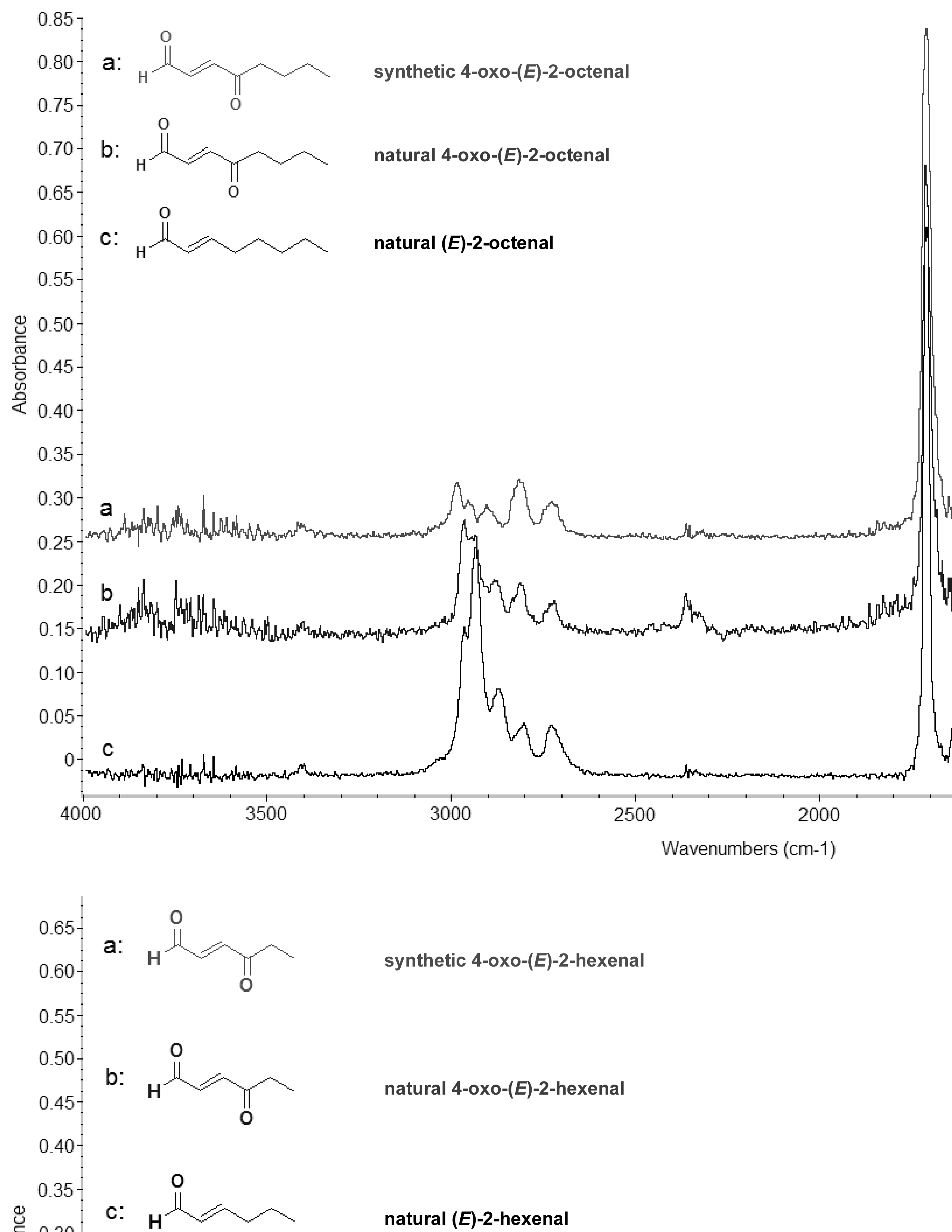

Supplement: Figure S1 — Infrared spectra of natural and synthetic odour components. Infrared spectra of (E)-2-hexenal, (E)-2-octenal, 4-oxo-(E)-2-hexenal and 4-oxo-(E)-2-octenal from C. lectularius extracts and synthetic reference compounds. Gas Chromatography-Fourier transform infrared spectroscopy (GC-FTIR) analyses were carried out on extracts and reference compounds using a GC (Agilent Technologies 7890A) equipped with a polar capillary column (FactorFour, VF-23ms; 30 m long, 0.25 mm inner diameter and 0.25 µm film thickness), coupled to a FTIR (Termo Fisher Nicolet 6700 FT-IR). The injector temperature was 250°C and the injector was splitless. The temperature was programmed at 50°C for 0 min, followed by a gradual increase of 10°C/min to reach a final temperature of 230°C for 10 min. IR data for the compounds: 4-oxo-(E)-2-hexenal: IR (vapour phase): ν (cm−1): 2987, 2818, 27301, 1712, 1106, 1048, 980 and 4-oxo-(E)-2-octenal: IR (vapour phase): ν (cm−1): 2967, 1715, 1102, 1073, 980. IR (neat): ν (cm−1): 2958, 2933, 2873, 1711, 1172, 1049. (TIF) [file pone.0018156.s001.tif]
